# Concomitant transcatheter closure of post-myocardial infarction ventricular septal defect and inferior wall aneurysm: case report

**DOI:** 10.1093/ehjcr/ytaa408

**Published:** 2020-11-27

**Authors:** Reda Abuelatta, Tarek Alrashidy, Fatma Taha, Hesham Abdo Naeim

**Affiliations:** Madinah Cardiac Center, Khaled Bin Waleed Street, PO 6167, Madinah, Saudi Arabia

**Keywords:** Acute myocardial infarction, Ventricular septum rupture, Amplatzer occluder devices, Ventricular aneurysm, Case report

## Abstract

**Background:**

The incidence of the post-infarct ventricular septal defect (VSD) is 0.17%. Surgical repair is the definitive treatment and percutaneous closure is an alternative in high-risk patients. We report a case of post-myocardial infarction inferior wall aneurysm associated with a large ventricular septal rupture, with a communication between the aneurysm and right ventricle. Successful percutaneous closure of both the aneurysm and the post-infarct (VSD) was performed using two Amplatzer septal occluder devices.

**Case summary:**

A 76-year-old man was referred to the clinic 2 weeks after an inferior wall myocardial infarction. A harsh, pansystolic murmur was appreciated on his left parasternal area and across the pericardium. An echocardiogram demonstrated a large, true aneurysm in the mid-cavity inferior wall. The inferior septum was ruptured and dissected, with a large, left-to-right shunt. The patient’s coronary angiography revealed a multi-vessel disease. The patient was considered as high surgical risk and thus transcatheter closure of both the post-infarct VSD and inferior wall aneurysm was recommended. We crossed the VSD from the venous side. An Amplatzer septal occluder (18 mm) was deployed to close the VSD completely. We crossed the aneurysm mouth from the arterial side. Another Amplatzer septal occluder (26 mm) was deployed with the large disc inside the aneurysm, sealing it with no more flow. After discharge from the intensive care unit, the patient underwent complete revascularization for his right coronary artery, left main artery, proximal left anterior descending artery, and ramus intermedius. At his 3-month follow-up, the patient remained well with reasonable exercise tolerance.

**Discussion:**

Percutaneous closure of a post-infarct VSD and aneurysm is an option for patients whose comorbidities preclude surgical repair and whose septal anatomy is favourable to device placement.

**Learning points**

• The echocardiographic and fluoroscopic diagnosis of concomitant left ventricular aneurysm and ventricular septal defect post-myocardial infarction is mandatory to guide percutaneous closure.

• The percutaneous closure of both post-infarct ventricular septal defect and inferior aneurysm by two Amplatzer septal occluder devices is feasible.

• The post-infarct ventricular septal rupture incidence is low post-primary PCI era, but the clinician should be alerted as the outcome of this complication is poor.

## Introduction

Post-infarct ventricular septal defect (VSD) and aneurysm are serious complications of acute myocardial infarction (MI). The incidence of post-infarct VSD has decreased from 1–2% following ST-segment elevation MI in the pre-reperfusion era^1^ to 0.17% following primary percutaneous coronary intervention (PCI).^2^ Surgical repair is the definitive treatment, but it is associated with high morbidity and mortality. Percutaneous closure is an alternative to surgery in high-risk patients.^2^ We report a patient with a post-MI inferior wall aneurysm associated with a large post-infarct VSD, with a communication between the aneurysm and right ventricle (RV). We report successful percutaneous closure of both the aneurysm and post-infarct VSD using two Amplatzer septal occluder (ASO) devices. We emphasize the importance of multimodality imaging for accurate diagnosis and management. To the best of our knowledge, this is the first case reported treating these two defects in the same session using two different devices percutaneously.

## Timeline

**Table:** 

2 weeks before admission	A 76-year-old man presented with ST-elevation inferior wall myocardial infarction and received thrombolytic therapy in a hospital without primary PCI facilities.
Presentation at admission	He presented with typical chest pain (exertional dyspnoea New York Heart Association Class III).
Examination at admission	The patient was haemodynamically stable. A harsh pansystolic murmur was appreciated on his left parasternal area.
Transthoracic echocardiogram and transoesophageal echocardiogram after admission	Echocardiogram demonstrated akinetic inferior wall and inferior septum with an ejection fraction (EF) of 45%. A large, true aneurysm was observed in the mid-inferior wall. The inferior septum was ruptured and dissected with a large left-to-right shunt.
Procedure done after 3 days from admission	The patient was considered a high surgical risk. Transcatheter closure of both the post-infarct ventricular septal defect and inferior wall aneurysm was done using two Amplatzer septal occluder devices.
6 days in intensive care unit (ICU)	The patient recovered uneventfully within 6 days in the ICU.
Coronary revascularization after discharge from ICU	The patient underwent complete revascularization of his right coronary artery, left main artery, proximal left anterior descending artery, and Ramus intermedius.
After 5 days	The patient discharged home in a good condition.
Follow-up after 3 months	The patient remained well with reasonable exercise tolerance. A repeat transthoracic echocardiography demonstrated no flow across the devices, with no residual shunt and an EF of 45%.

## Case presentation

A 76-year-old man presented with typical chest pain and ST-elevation inferior MI. He was a smoker with diabetes and chronic kidney disease Class 3a with a glomerular filtration rate (GFR) 48 mL/min/m^2^. The hospital had no coronary intervention facilities, so he received thrombolytic therapy. Two weeks later, he presented to our emergency department with typical chest pain and exertional dyspnoea (New York Heart Association Class III). He was on dual antiplatelet therapy, Bisoprolol, Atorvastatin, and Isosorbide dinitrates. There was no echocardiogram report with the patient.

Auscultation confirmed a harsh pansystolic murmur V/VI on his left parasternal area and across the precordium. He had bilateral, fine basal crepitations. His heart rate was 88 b.p.m. and blood pressure was 115/67 mmHg.

**Figure 1:**
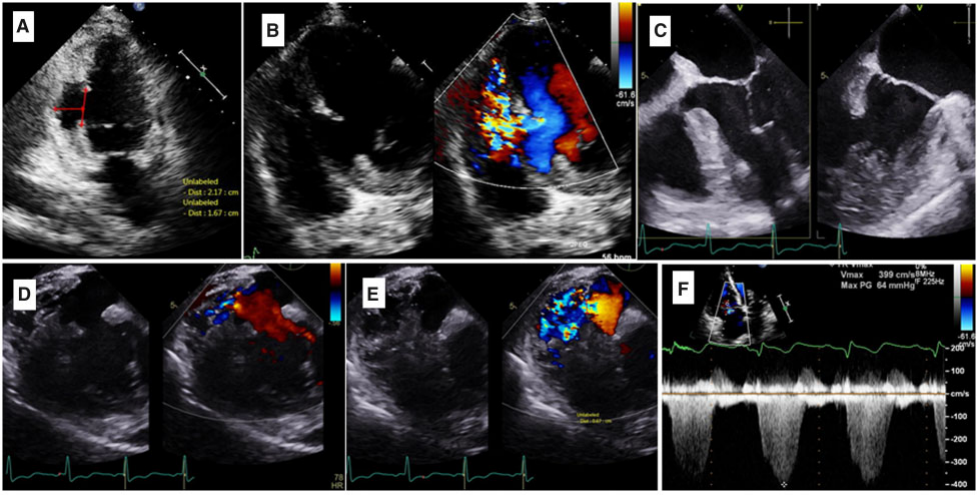
(*A*) Transthoracic echocardiogram apical two-chamber view showing inferior wall true aneurysm. (*B*) Transthoracic echocardiogram modified apical view showing inferior septum ruptured and dissected with left-to-right shunt. (*C*) Transoesophageal echocardiogram X-plane showing the inferior wall aneurysm. (*D*) Transoesophageal echocardiogram transgastric short-axis view in diastole showing the inferior wall aneurysm with a wide neck. (*E*) Transoesophageal echocardiogram transgastric short-axis view in systole showing the inferior wall aneurysm communication with right ventricle. (*F*) Mild tricuspid regurgitation with an estimated systolic pulmonary artery pressure of 75 mmHg.

**Figure 2:**
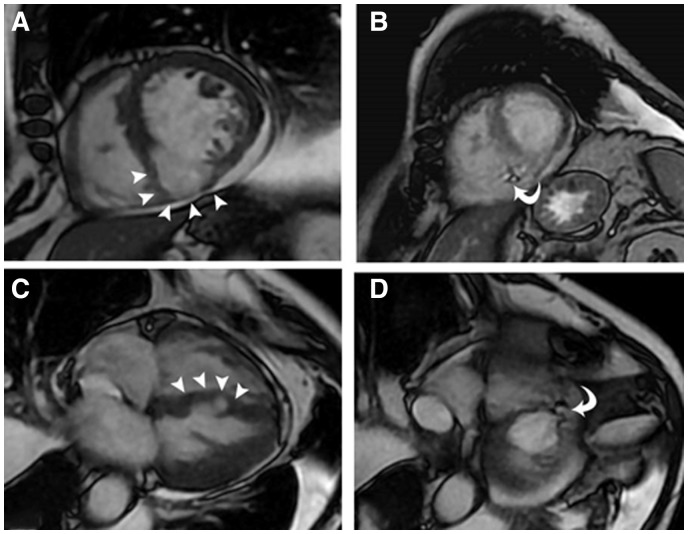
Cardiac magnetic resonance imaging: Balanced steady-state gradient echo cine images [short-axis plane (*A, B*), four-chamber plane (*C, D*)]. The LV shows a mid-inferior and inferoseptal wide-neck -true aneurysm (arrow heads in *A, C*) with a focal area of ventricular septal rupture in the inferior aspect of the aneurysm with a turbulent high-velocity flow across it (curved arrows in *B, D*).

Transthoracic echocardiogram (TTE) and transoesophageal echocardiogram (TOE) demonstrated an akinetic inferior wall and inferior septum with an ejection fraction (EF) of 45%. A large, true aneurysm in the mid-cavity inferior wall was identified by its wide neck and contracting muscle layer (*Figure 1A* and Supplementary material online, *Video S1*). The inferior septum was ruptured and dissected with a large, left-to-right shunt (*Figure 1B* and Supplementary material online, *Video S2*). A TOE X-plane transgastric short-axis view in diastole showed an inferior wall aneurysm with a wide neck of 2 cm (*Figure 1C* and *D*). The aneurysm cavity connected to the RV through the ruptured part of the inferior septum, creating an LV-to-RV-to-aneurysm communication (*Figure 1E* and Supplementary material online, *Video S3*). We noted mild tricuspid regurgitation with an estimated systolic pulmonary artery pressure of 75 mmHg. RV function was mildly reduced (tricuspid annular plane systolic excursion of 1.6 cm). Cardiac magnetic resonance imaging showed non-viable inferior segments, and a 4.2 cm × 4.3 cm × 5.8 cm cavitary lesion. The LV showed a mid-inferior and inferoseptal wide-neck true aneurysm (*Figure 2A* and *C*) with a focal area of ventricular septal rupture in the inferior aspect of the aneurysm with a turbulent high-velocity flow across it (*Figure 2B* and *D*). The patient’s coronary angiography displayed tight proximal and middle right coronary artery lesions, as well as a severe ostial left main, a severe mid-left anterior descending artery lesion, and severe ramus intermedius disease.

The patient was considered high surgical risk because of his age, general condition, renal impairment, and difficult anatomy of the post-infarct VSD and aneurysm. Transcatheter closure of the post-infarct VSD and inferior wall aneurysm was performed. Because of the two different pathologies, we closed the VSD first using an antegrade approach from the venous side. Next, we closed the neck of the aneurysm using a retrograde approach from the arterial side. A Terumo guidewire (260 cm) was introduced from the right common femoral vein. The wire passed from the RV to the inferior wall aneurysm to the LV and finally to the aorta. We then exchanged the Terumo guidewire with an Amplatzer stiff guidewire (260 cm).

**Figure 3:**
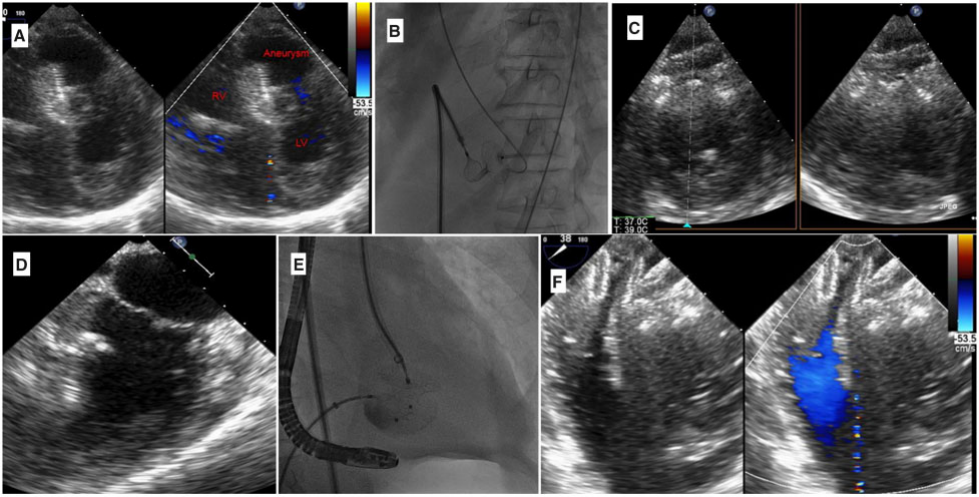
(*A*) Transoesophageal echocardiogram showing an 18-mm Amplatzer septal occluder device used to close the aneurysm to RV communication. (*B*) Fluoroscopy showing the first deployed 18-mm device. (*C*) Transoesophageal echocardiogram transgastric short-axis view showing the 26-mm Amplatzer septal occluder device closing the mouth of the aneurysm. (*D*) Transoesophageal echocardiogram two-chamber view showing the 26-mm Amplatzer septal occluder device closing the mouth of the aneurysm. (*E*) Fluoroscopy showing the two deployed devices connected by cables: one from the femoral vein and the other from the femoral artery. (*F*) Transoesophageal echocardiogram showing the two devices, with the 26-mm device resting on the 18-mm one, no colour flow seen.

We used an 8 French Amplatzer delivery sheath to deploy an 18-mm ASO (AGA Medical Corporation, USA) to close the VSD completely (*Figure 3A* and *B*, and Supplementary material online, *Video S4*). We selected the size based on TOE measurements. The RV-to-aneurysm communication measured 14 mm, so we oversized the ASO device by 4 mm to fit inside the defect. The aneurysm was engaged in retrograde from the LV using a 6 French PG catheter introduced from the right common femoral artery and over the Terumo guidewire (260 cm), which was then replaced by a Confida stiff guidewire (Medtronic) of the same length. We cannulated the inferior aneurysm mouth with a Torque-Vue 10 F/45 delivery sheath. The ASO (26 mm) device was deployed with a large disc in the aneurysm (*Figure 3C–3E*, and Supplementary material online, *Video S5*). We selected the size based on the TOE measurement. The aneurysm mouth measured 20 mm, so we increased the ASO length by 6 mm to fit inside the aneurysm. We assured enough distance between the aneurysm mouth and the mitral valve so that the deployed disc would not affect the mitral valve. Both devices were established with the aneurysm device resting on the post-infarct VSD device (*Figure 3F* and Supplementary material online, *Video S6*).

**Figure 4:**
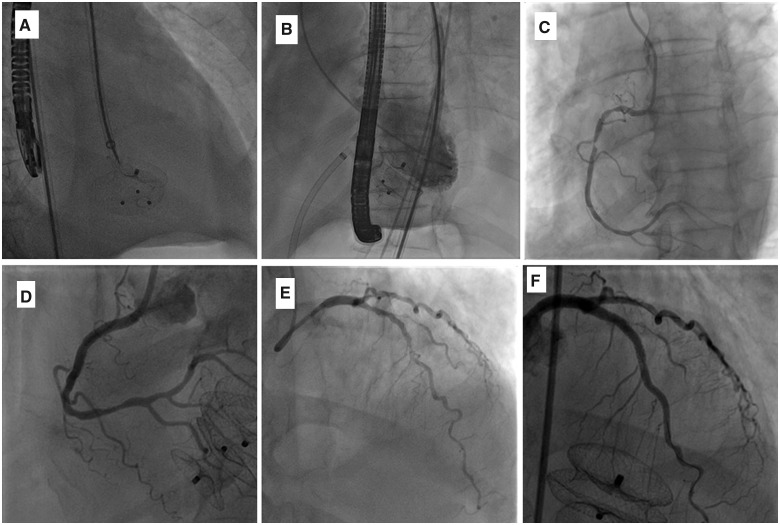
(*A*) The 18-mm device, released first. (*B*) The 26-mm device with left ventricle injection, released second showing more flow to the right ventricle or aneurysm. (*C*) Critical right coronary artery proximal and middle lesions. (*D*) Right coronary artery after revascularization. (*E*) Critical left anterior descending artery and LCX lesions: (*F*) Left anterior descending artery and LCX after revascularization. LCX, left circumflex.

**Figure 5:**
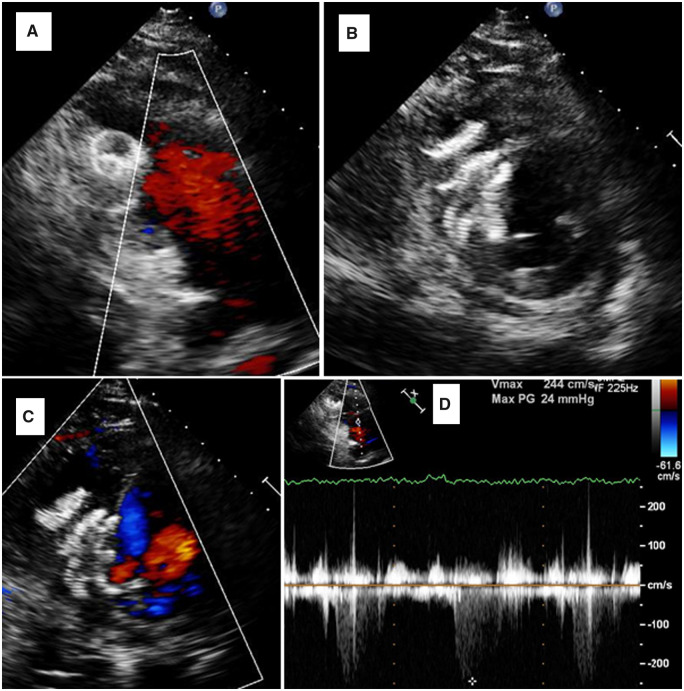
(A) Transthoracic echocardiogram at follow-up, right ventricular inflow view showing the right ventricular disc of the 18-mm device. (*B*) Transthoracic echocardiogram short-axis view showing both devices, with the aneurysmal cavity closed. (*C*) No more colour flow to aneurysm or right ventricle. (*D*) Estimated systolic pulmonary artery pressure of 35 mmHg.

Left ventricular (LV) injection and TOE showed complete occlusion of the post-infarct VSD and aneurysm. Both devices established contact and showed the angulated relation of their two different planes (*Figure 4A* and *B*, and Supplementary material online, *Video S7*). The akinetic inferior wal procedure and fluoroscopy times were 60 min and 25 min, respectively, and the total contrast amount was 40 mL. The patient recovered uneventfully within 6 days in the intensive care unit. As EF was 45%, we selected to do full revascularization. The patient underwent complete revascularization for his right coronary artery, left main artery, proximal left anterior descending artery, and ramus intermedius (*Figure 4C–F*). The patient was discharged after 14 days. At his 3-month follow-up, he remained well with reasonable exercise tolerance, NYHA Class I. A repeat transthoracic echocardiogram demonstrated no flow across the devices, no residual shunt, and a left ventricular ejection fraction of 45%, akinetic inferior wall, and inferior septum (*Figure 5A–C*, and Supplementary material online, *Video S8*). The systolic pulmonary artery pressure improved to 35 mmHg (*Figure 4D*). His renal function remained stable at follow-up with GFR 46 mL/min/m^2^.

## Discussion

We report a successful closure of a post-infarct VSD and an inferior wall aneurysm in the same session at 17 days after an inferior wall MI. A literature review of 176 patients in 10 years concluded that the device closure of post-infarct VSD is feasible and safe in selected patients.^3^ The success rate is 50–86%, and the 30-day mortality rate is 20–39% if the closure is performed at 14 days or more after MI. The most important risk factors associated with mortality are the presence of cardiogenic shock and closure in the acute phase (<14 days).^3^ A large surgical series (*n* = 2876) from the Society of Thoracic Surgeons National Database reports an overall operative death rate of 42.9%.^4^ In the UK, the in-hospital mortality rate among 53 patients with post-infarct VSD at 11 centres was 34%, even after successful procedures. Although the operative mortality is reduced by delaying surgery, this may be because many patients do not survive long enough on medical therapy. Data suggest that patients with post-infarction VSD treated with percutaneous device closure relatively early do well, especially if they survive to discharge.^5^ Another study found that among 32 patients with post-infarct VSD, only 22% of defects were confined to the septum; moreover, right coronary artery MI resulting in inferoseptal wall VSD was mostly associated with intramyocardial dissection and involvement of the free wall.^6^

Although the 2013 American College of Cardiology and American Heart Association guidelines recommend emergent surgical repair of post-infarct VSD, regardless of haemodynamic status, the timing of surgery remains controversial.^7^ The 2017 European Society of Cardiology guidelines promote delayed elective repair in patients who initially respond to aggressive conservative management.^8^ Transcatheter closure of post-infarct VSD has been suggested as a viable alternative to surgical repair.^9^ One report also demonstrates occluder implantation in a post-infarct VSD adjacent to an aneurysm.^10^ Successful closure of a large, post-infarct VSD (≥15 mm) with a large aneurysm has not been previously reported.

In this case, the post-infarct VSD and aneurysm were large, with an insufficient muscular rim at the mouth of the aneurysm. The 18-mm ASO occluder was well positioned within the post-infarct VSD, and the 26-mm ASO was enough to close the whole aneurysm and its opening while pressing against the first device, with no residual shunt. This case report thus contributes to the understanding of echocardiographic and fluoroscopic diagnoses of concomitant post-MI left ventricular aneurysm and VSD. It also highlights the feasibility and technique of percutaneous closure of both post-infarct VSD and inferior aneurysm using two ASO devices. Finally, although post-infarct ventricular septal rupture incidence is low in the post-primary percutaneous coronary intervention era, the clinician should be alerted, as the outcome of this complication is poor. In the Covid-19 era, where we are seeing more late presentation STEMIs, the post-infarction VSD incidence may be increased and necessitate early intervention.

## Conclusion

The management of post-MI mechanical complications, including post-infarct VSD and aneurysm, is complicated and requires substantial critical care, imaging, intervention, and surgical expertise. Percutaneous closure is an option for patients whose comorbidities preclude surgical repair and whose septal anatomy is favourable to device placement. 

## Lead author biography

**Figure:**
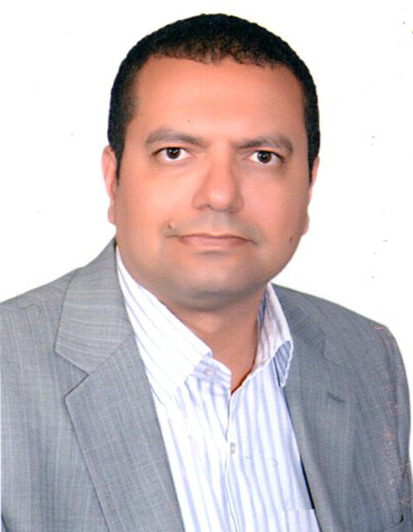


Dr Hesham Naeim, MD, FASE graduated from Faculty of Medicine, Al-Azhar University at December 1997. Granted M.Sc. degree in cardiovascular diseases at December 2002. Granted MD degree in cardiovascular diseases and interventions 2006. Diplomate - Adult Comprehensive Echocardiography from National Board of Echocardiography, United States at June 2014. Resident and Assistant Lecturer of Cardiology, in Al-Azhar University hospitals from June 1997 to February 2006. Cardiology consultant in MNH Saudi Arabia from January 2007 till April 2013. Adult cardiology consultant in Madinah Cardiac Center Saudi Arabia from June 2013 till now. Dr Hesham is expert in Echocardiography in structural heart disease.

## Supplementary material

Supplementary material is available at *European Heart Journal - Case Reports* online.

## Supplementary Material

ytaa408_Supplementary_Data
