# Supplementary material for: Concomitant transcatheter closure of post-myocardial infarction ventricular septal defect and inferior wall aneurysm: case report
Source: Eur Heart J Case Rep. 2020 Nov 27;4(6):1–7. doi: 10.1093/ehjcr/ytaa408 (PMC7793211; doi:10.1093/ehjcr/ytaa408)
Supplement: ytaa408_Supplementary_Data [file ytaa408_supplementary_data.zip › ytaa408-suppl_data/EHJ-CR_Slide_Set.pptx]

## Slide 1
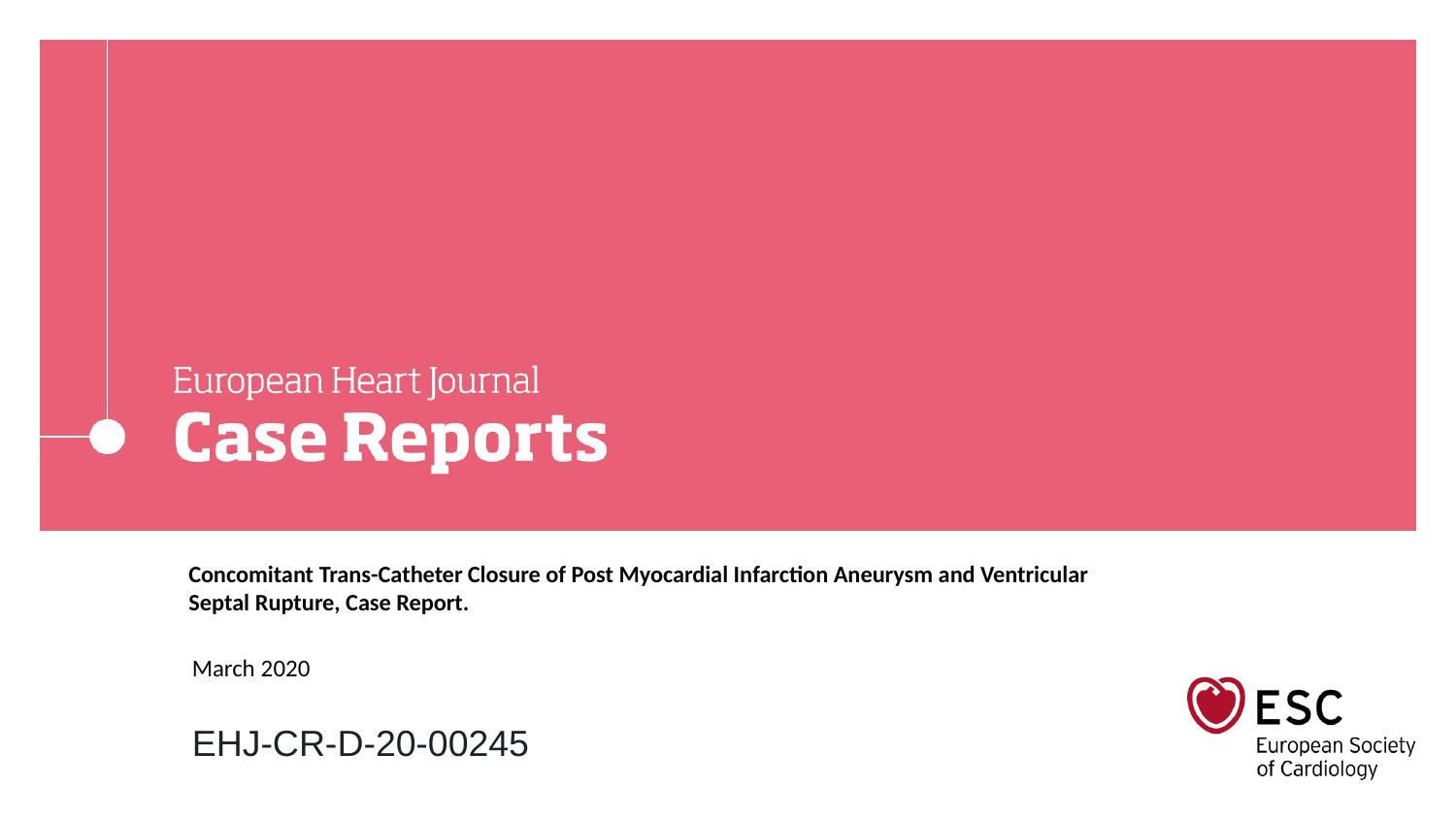

# Concomitant Trans-Catheter Closure of Post Myocardial Infarction Aneurysm and Ventricular Septal Rupture, Case Report.
March 2020
EHJ-CR-D-20-00245

## Slide 2
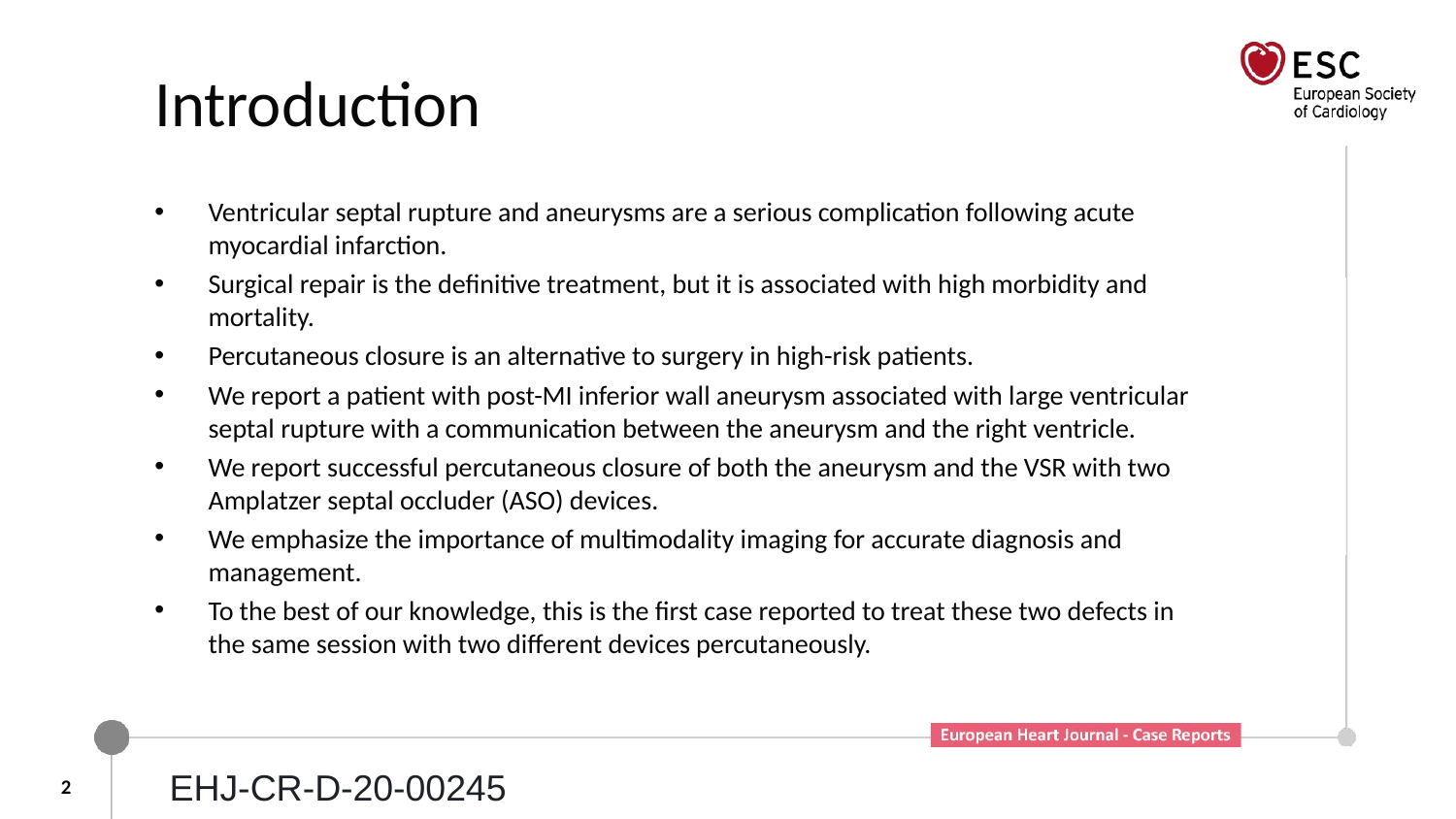

# Introduction
Ventricular septal rupture and aneurysms are a serious complication following acute myocardial infarction.
Surgical repair is the definitive treatment, but it is associated with high morbidity and mortality.
Percutaneous closure is an alternative to surgery in high-risk patients.
We report a patient with post-MI inferior wall aneurysm associated with large ventricular septal rupture with a communication between the aneurysm and the right ventricle.
We report successful percutaneous closure of both the aneurysm and the VSR with two Amplatzer septal occluder (ASO) devices.
We emphasize the importance of multimodality imaging for accurate diagnosis and management.
To the best of our knowledge, this is the first case reported to treat these two defects in the same session with two different devices percutaneously.
2
EHJ-CR-D-20-00245

## Slide 3
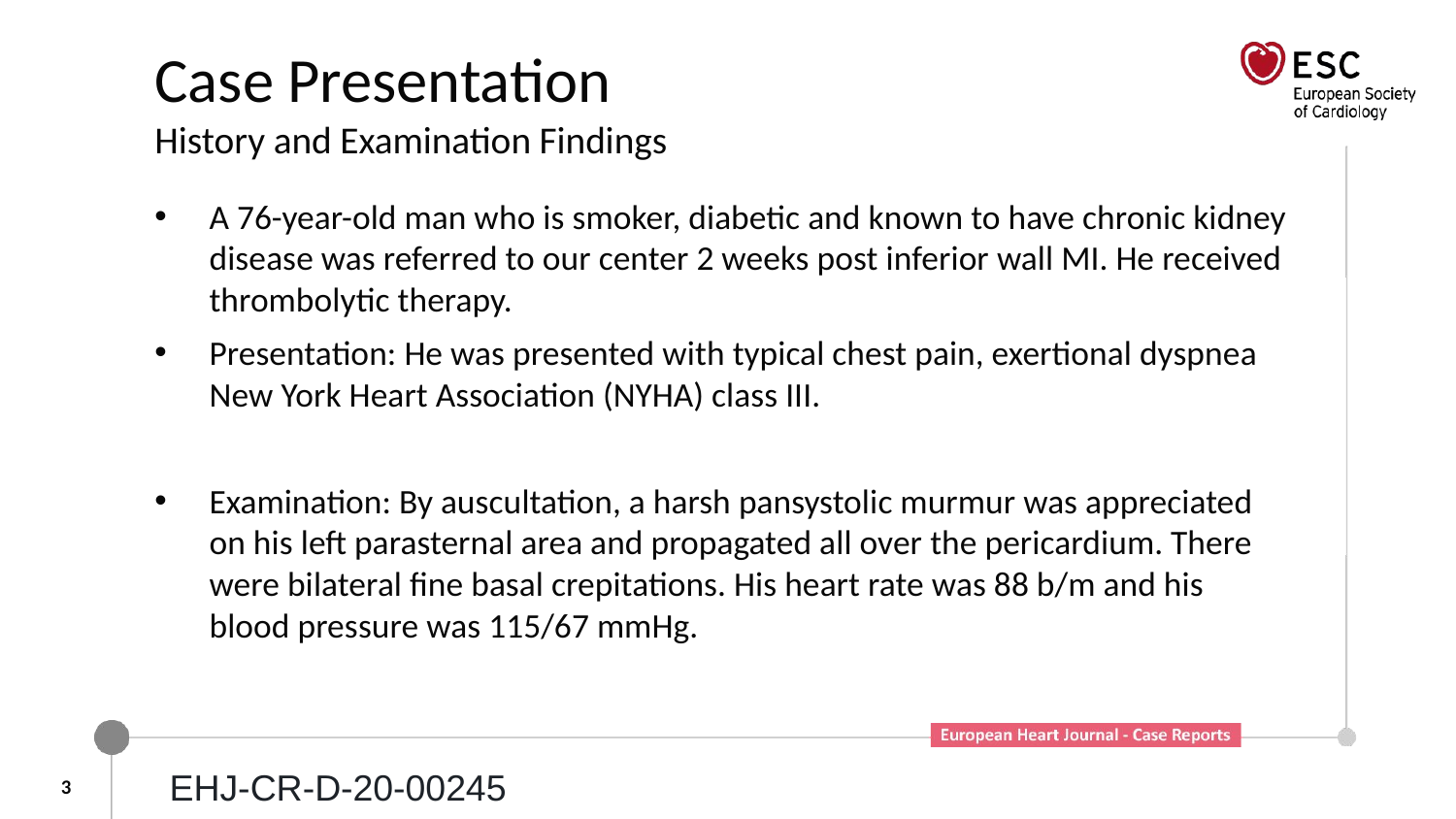

# Case Presentation History and Examination Findings
A 76-year-old man who is smoker, diabetic and known to have chronic kidney disease was referred to our center 2 weeks post inferior wall MI. He received thrombolytic therapy.
Presentation: He was presented with typical chest pain, exertional dyspnea New York Heart Association (NYHA) class III.
Examination: By auscultation, a harsh pansystolic murmur was appreciated on his left parasternal area and propagated all over the pericardium. There were bilateral fine basal crepitations. His heart rate was 88 b/m and his blood pressure was 115/67 mmHg.
3
EHJ-CR-D-20-00245

## Slide 4
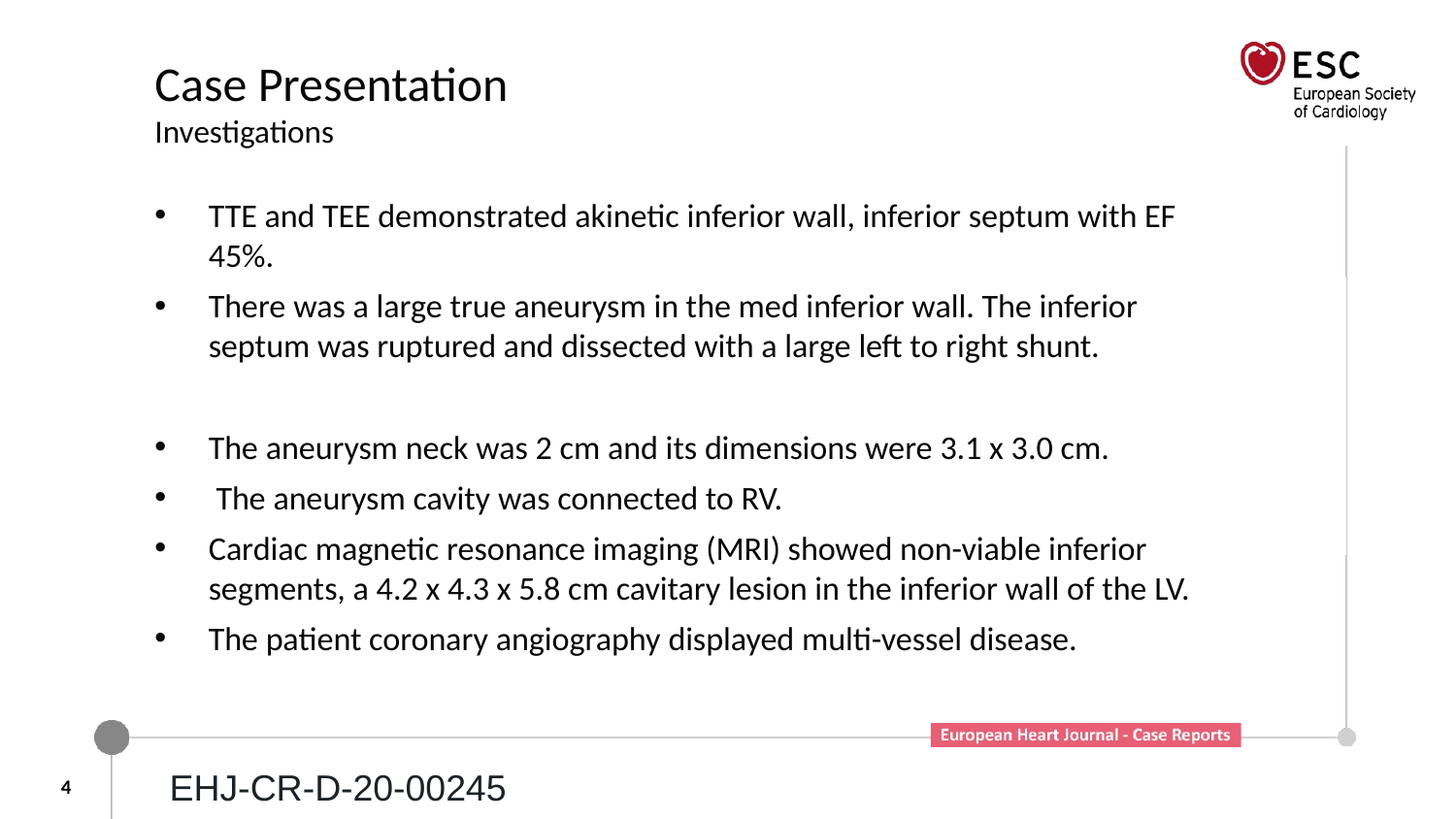

# Case Presentation Investigations
TTE and TEE demonstrated akinetic inferior wall, inferior septum with EF 45%.
There was a large true aneurysm in the med inferior wall. The inferior septum was ruptured and dissected with a large left to right shunt.
The aneurysm neck was 2 cm and its dimensions were 3.1 x 3.0 cm.
 The aneurysm cavity was connected to RV.
Cardiac magnetic resonance imaging (MRI) showed non-viable inferior segments, a 4.2 x 4.3 x 5.8 cm cavitary lesion in the inferior wall of the LV.
The patient coronary angiography displayed multi-vessel disease.
4
EHJ-CR-D-20-00245

## Slide 5
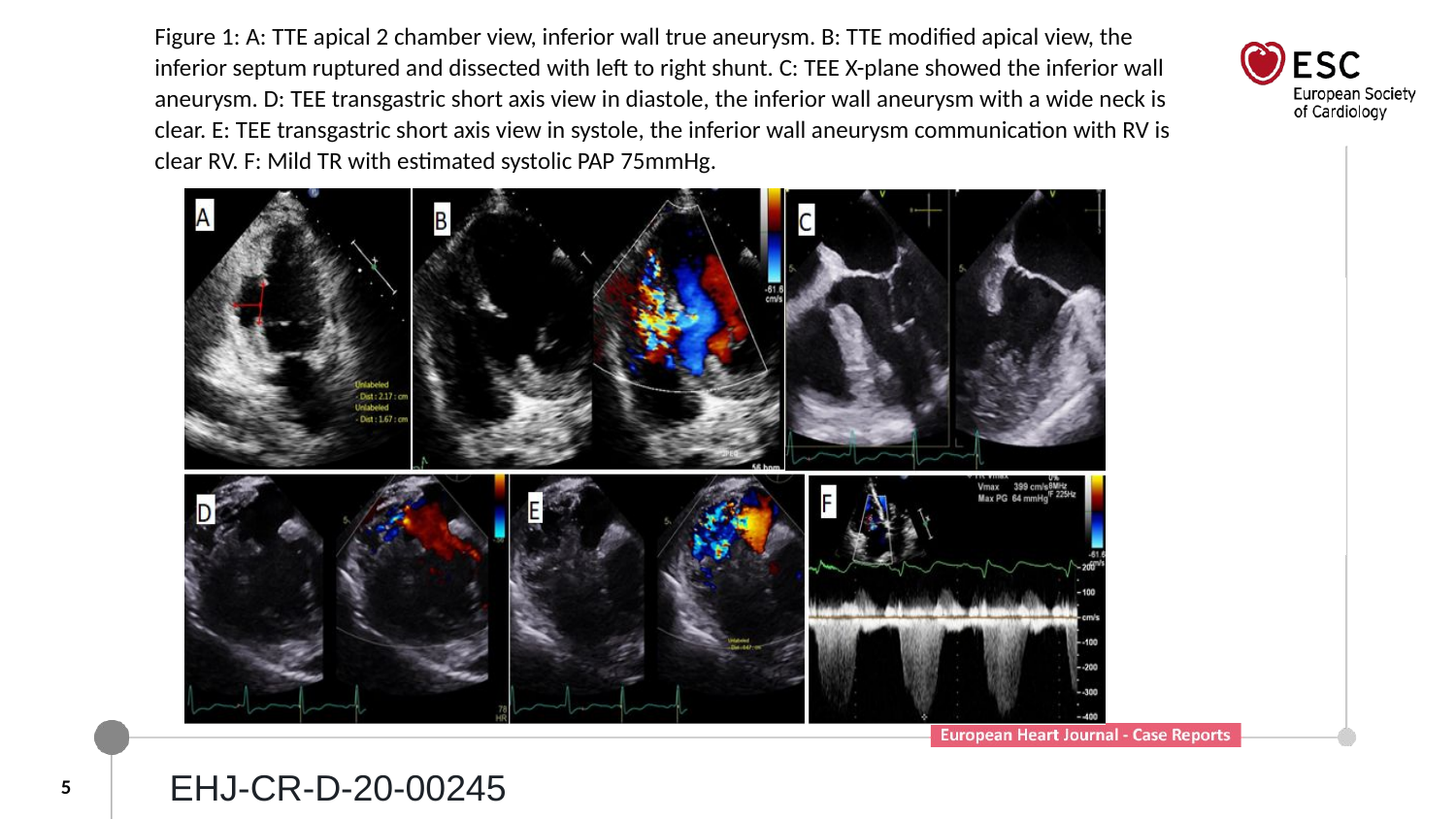

# Figure 1: A: TTE apical 2 chamber view, inferior wall true aneurysm. B: TTE modified apical view, the inferior septum ruptured and dissected with left to right shunt. C: TEE X-plane showed the inferior wall aneurysm. D: TEE transgastric short axis view in diastole, the inferior wall aneurysm with a wide neck is clear. E: TEE transgastric short axis view in systole, the inferior wall aneurysm communication with RV is clear RV. F: Mild TR with estimated systolic PAP 75mmHg.
5
EHJ-CR-D-20-00245

## Slide 6
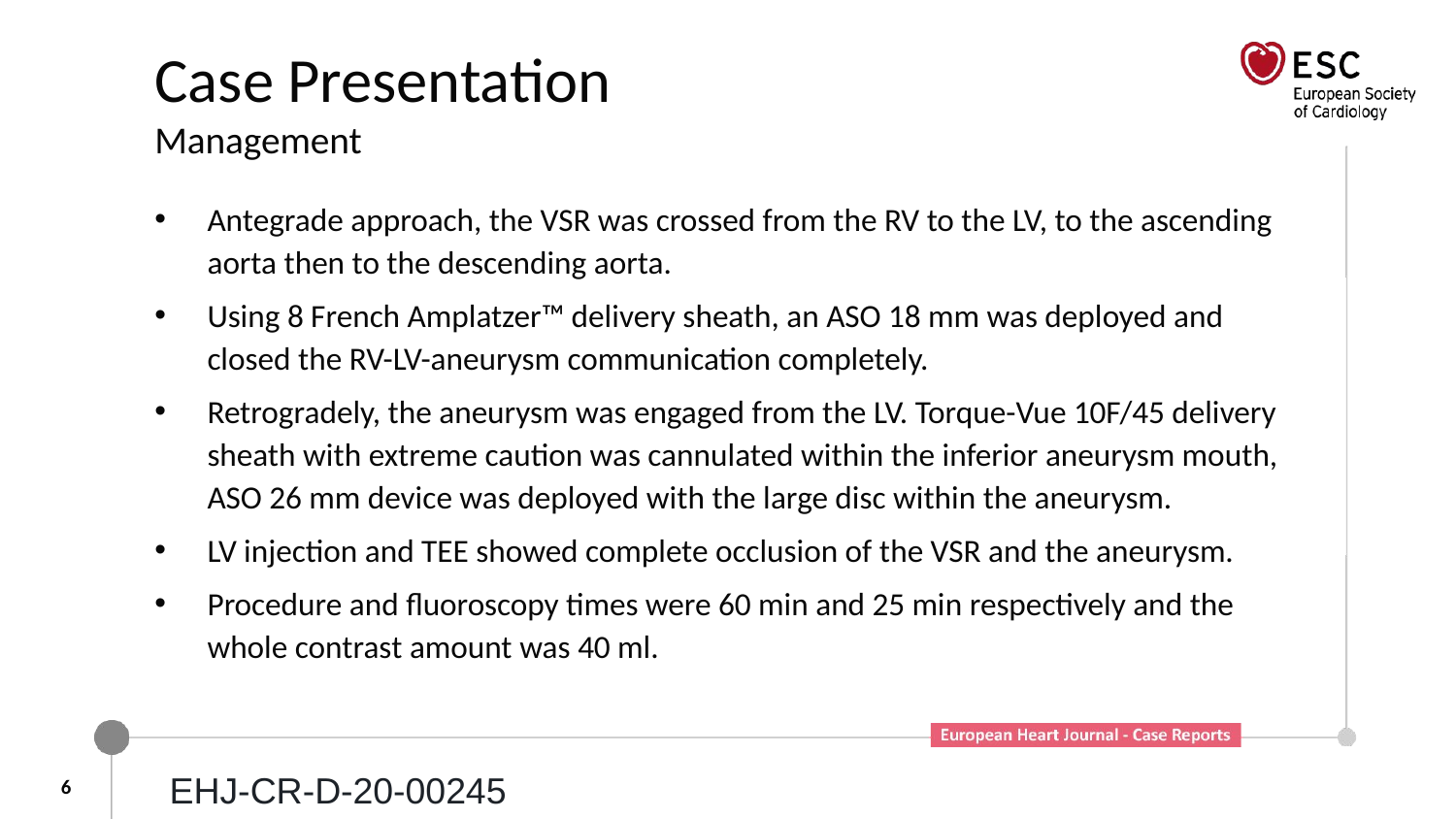

# Case Presentation Management
Antegrade approach, the VSR was crossed from the RV to the LV, to the ascending aorta then to the descending aorta.
Using 8 French Amplatzer™ delivery sheath, an ASO 18 mm was deployed and closed the RV-LV-aneurysm communication completely.
Retrogradely, the aneurysm was engaged from the LV. Torque-Vue 10F/45 delivery sheath with extreme caution was cannulated within the inferior aneurysm mouth, ASO 26 mm device was deployed with the large disc within the aneurysm.
LV injection and TEE showed complete occlusion of the VSR and the aneurysm.
Procedure and fluoroscopy times were 60 min and 25 min respectively and the whole contrast amount was 40 ml.
6
EHJ-CR-D-20-00245

## Slide 7
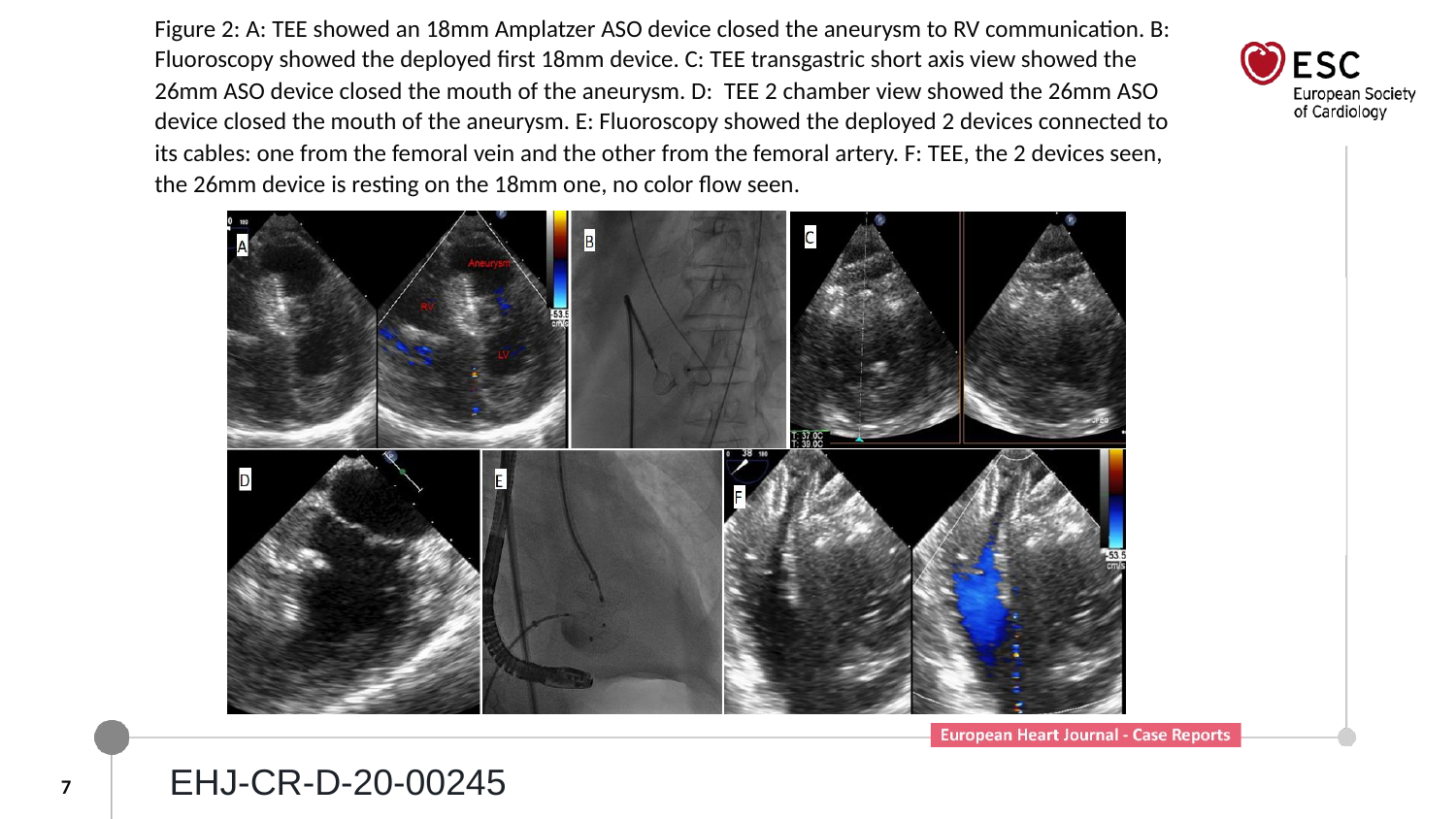

# Figure 2: A: TEE showed an 18mm Amplatzer ASO device closed the aneurysm to RV communication. B: Fluoroscopy showed the deployed first 18mm device. C: TEE transgastric short axis view showed the 26mm ASO device closed the mouth of the aneurysm. D: TEE 2 chamber view showed the 26mm ASO device closed the mouth of the aneurysm. E: Fluoroscopy showed the deployed 2 devices connected to its cables: one from the femoral vein and the other from the femoral artery. F: TEE, the 2 devices seen, the 26mm device is resting on the 18mm one, no color flow seen.
EHJ-CR-D-20-00245
7

## Slide 8
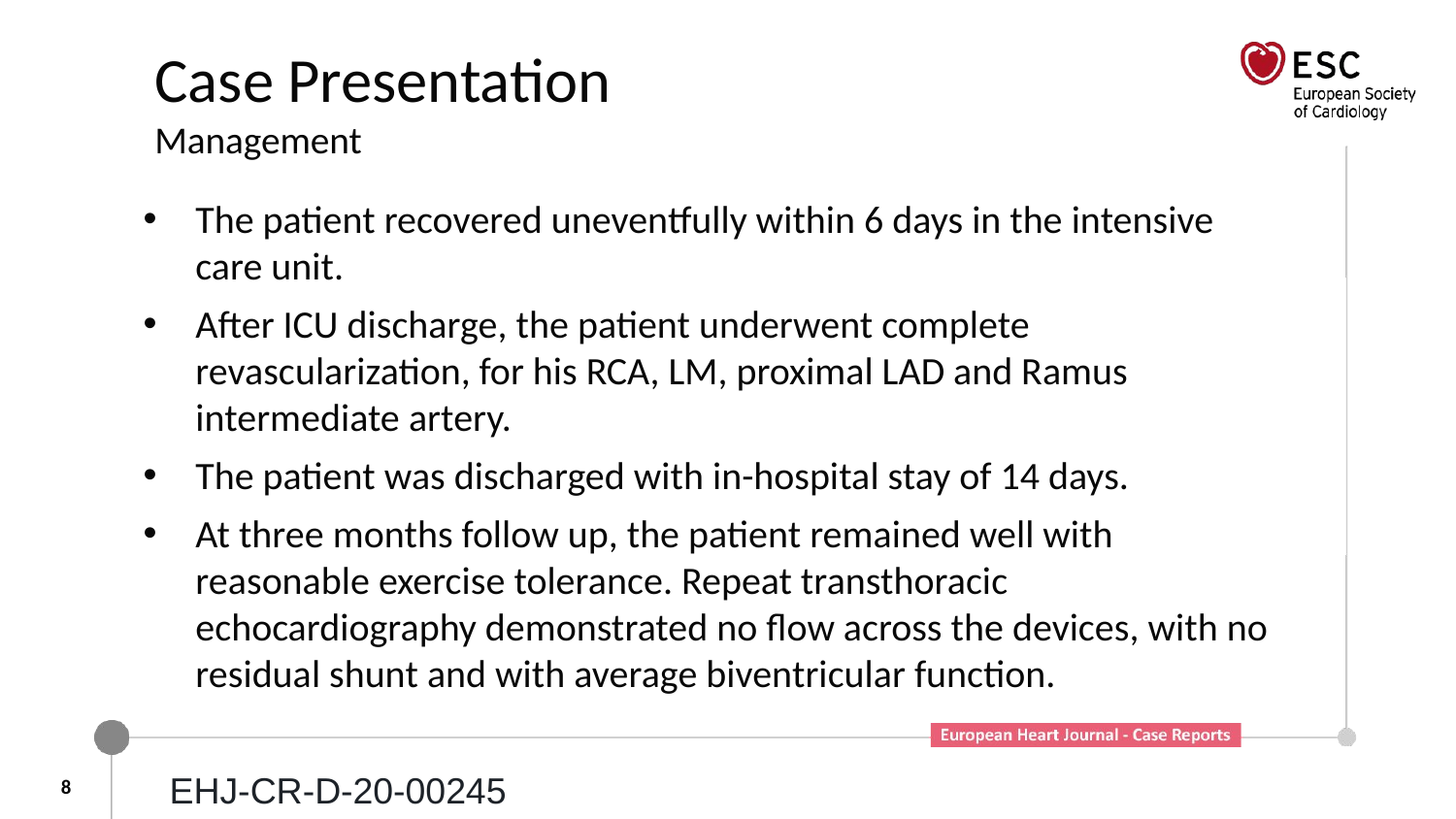

# Case Presentation Management
The patient recovered uneventfully within 6 days in the intensive care unit.
After ICU discharge, the patient underwent complete revascularization, for his RCA, LM, proximal LAD and Ramus intermediate artery.
The patient was discharged with in-hospital stay of 14 days.
At three months follow up, the patient remained well with reasonable exercise tolerance. Repeat transthoracic echocardiography demonstrated no flow across the devices, with no residual shunt and with average biventricular function.
8
EHJ-CR-D-20-00245

## Slide 9
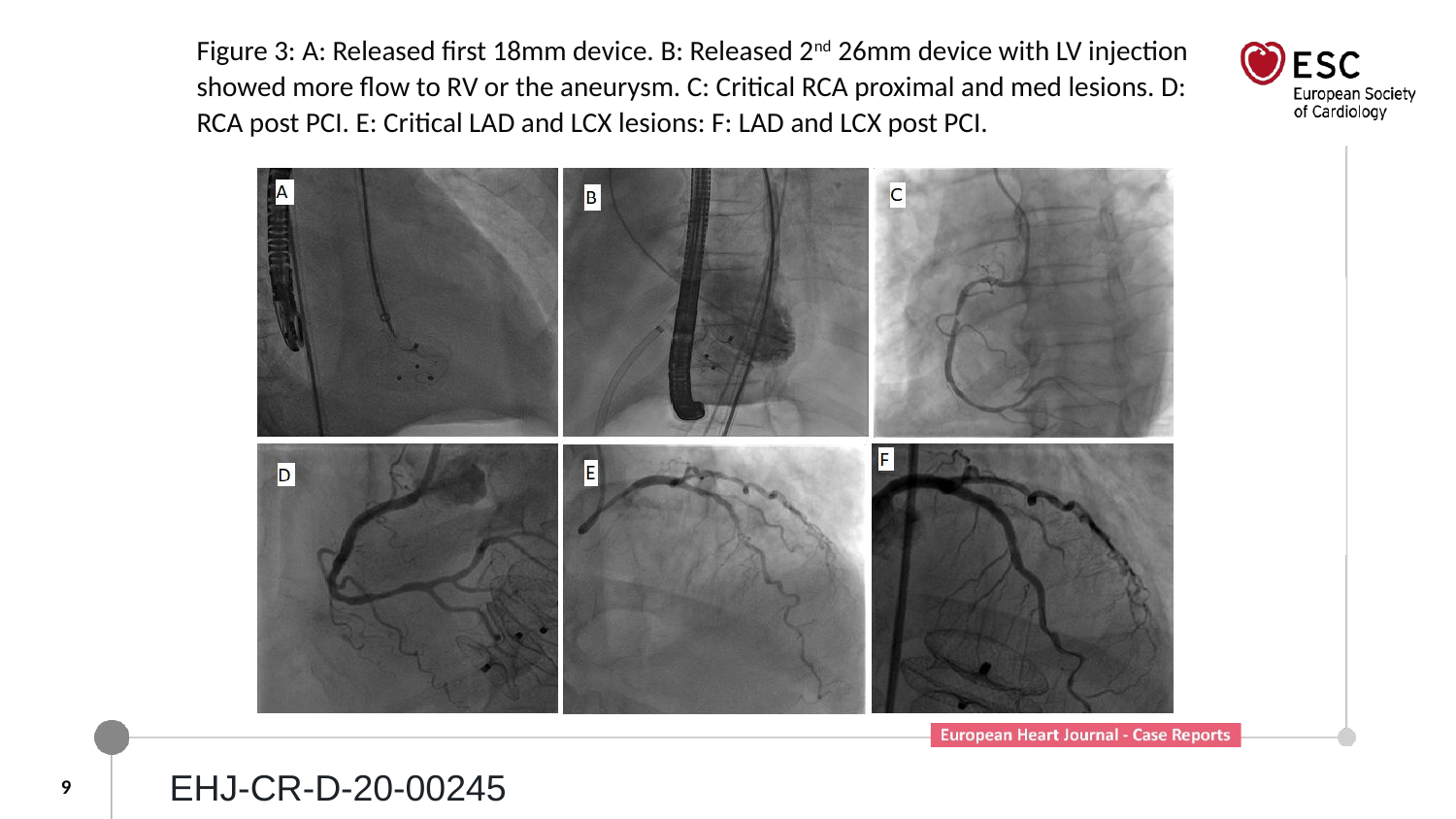

# Figure 3: A: Released first 18mm device. B: Released 2nd 26mm device with LV injection showed more flow to RV or the aneurysm. C: Critical RCA proximal and med lesions. D: RCA post PCI. E: Critical LAD and LCX lesions: F: LAD and LCX post PCI.
9
EHJ-CR-D-20-00245

## Slide 10
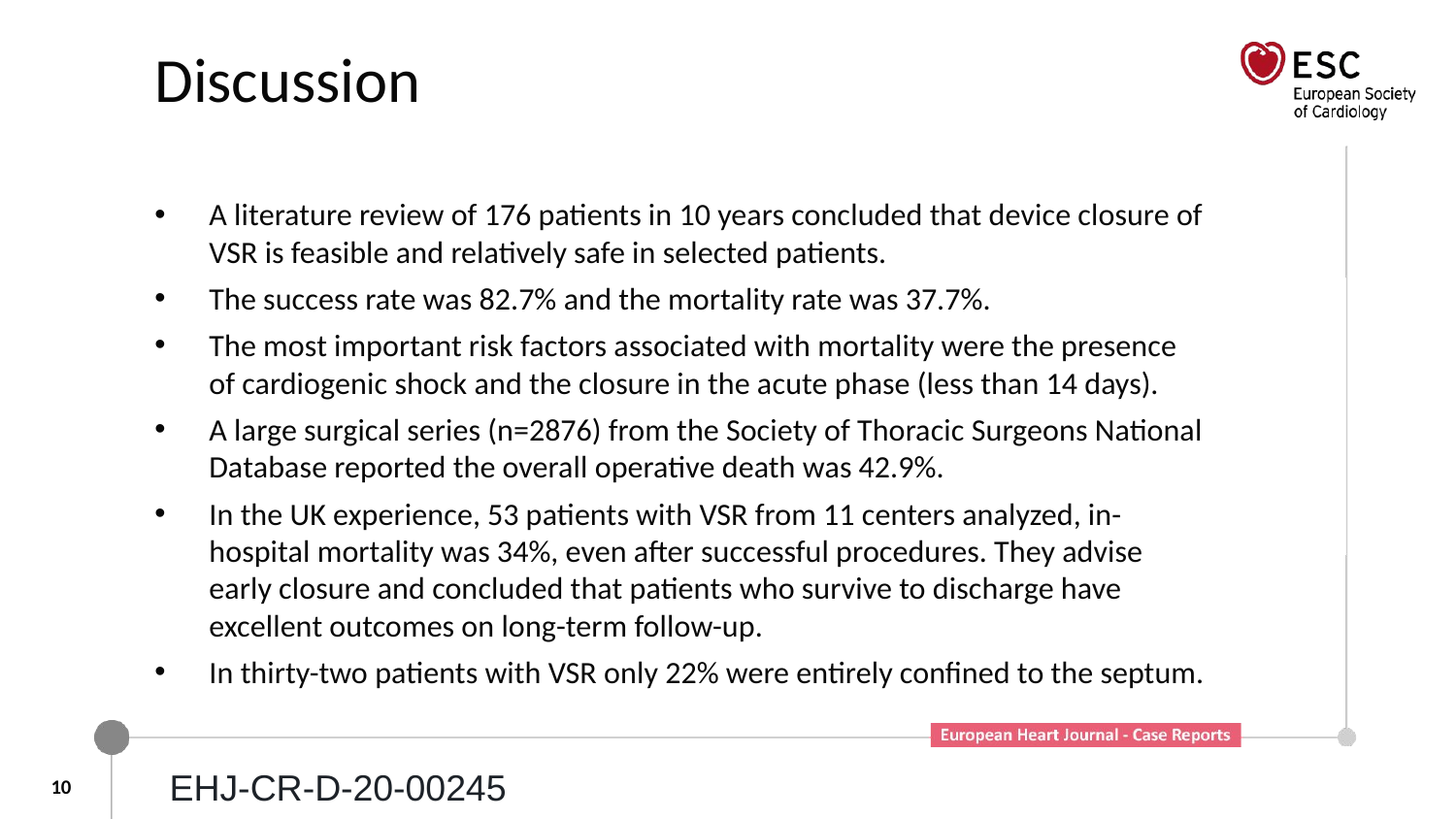

# Discussion
A literature review of 176 patients in 10 years concluded that device closure of VSR is feasible and relatively safe in selected patients.
The success rate was 82.7% and the mortality rate was 37.7%.
The most important risk factors associated with mortality were the presence of cardiogenic shock and the closure in the acute phase (less than 14 days).
A large surgical series (n=2876) from the Society of Thoracic Surgeons National Database reported the overall operative death was 42.9%.
In the UK experience, 53 patients with VSR from 11 centers analyzed, in-hospital mortality was 34%, even after successful procedures. They advise early closure and concluded that patients who survive to discharge have excellent outcomes on long-term follow-up.
In thirty-two patients with VSR only 22% were entirely confined to the septum.
10
EHJ-CR-D-20-00245

## Slide 11
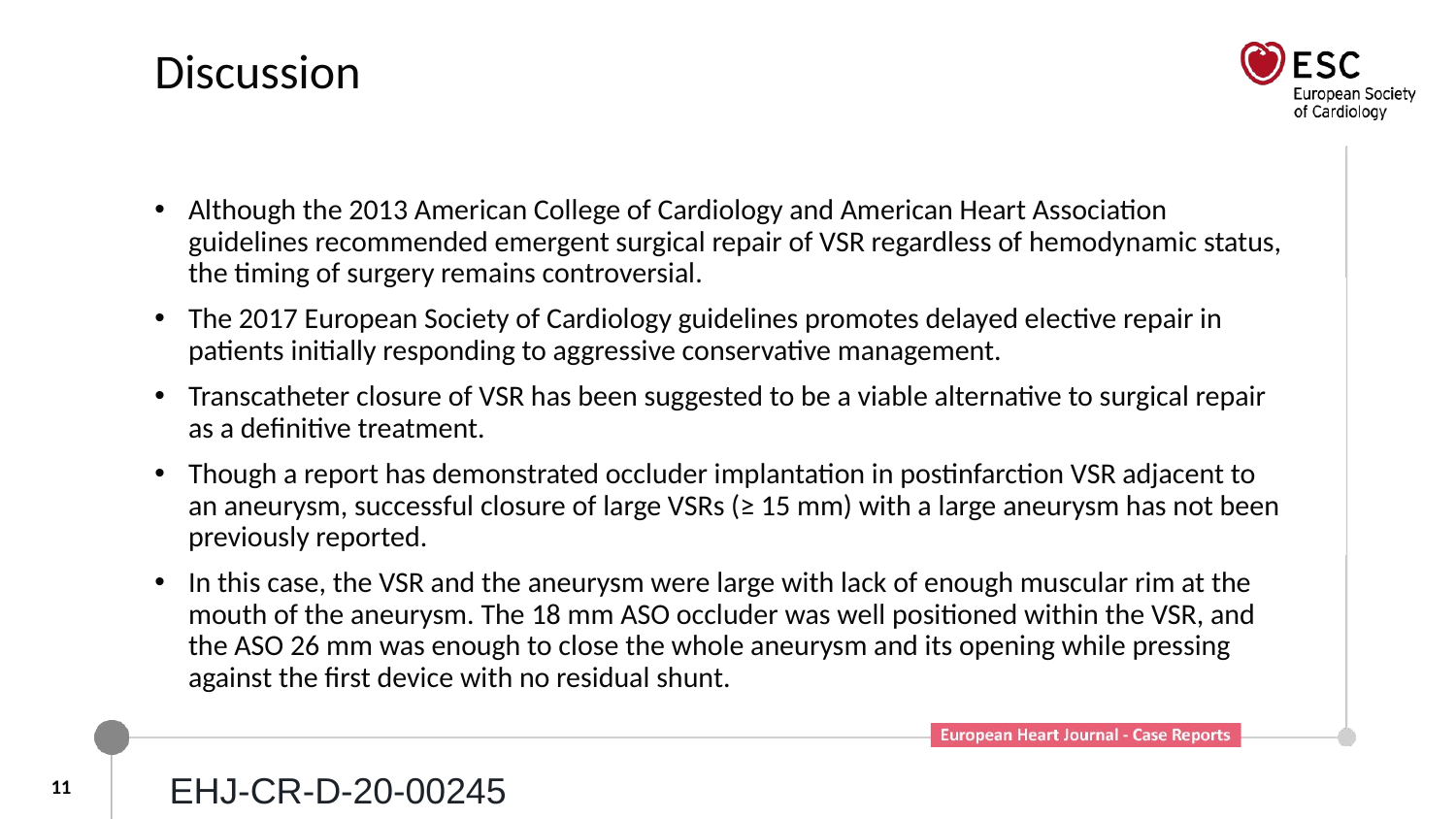

# Discussion
Although the 2013 American College of Cardiology and American Heart Association guidelines recommended emergent surgical repair of VSR regardless of hemodynamic status, the timing of surgery remains controversial.
The 2017 European Society of Cardiology guidelines promotes delayed elective repair in patients initially responding to aggressive conservative management.
Transcatheter closure of VSR has been suggested to be a viable alternative to surgical repair as a definitive treatment.
Though a report has demonstrated occluder implantation in postinfarction VSR adjacent to an aneurysm, successful closure of large VSRs (≥ 15 mm) with a large aneurysm has not been previously reported.
In this case, the VSR and the aneurysm were large with lack of enough muscular rim at the mouth of the aneurysm. The 18 mm ASO occluder was well positioned within the VSR, and the ASO 26 mm was enough to close the whole aneurysm and its opening while pressing against the first device with no residual shunt.
11
EHJ-CR-D-20-00245

## Slide 12
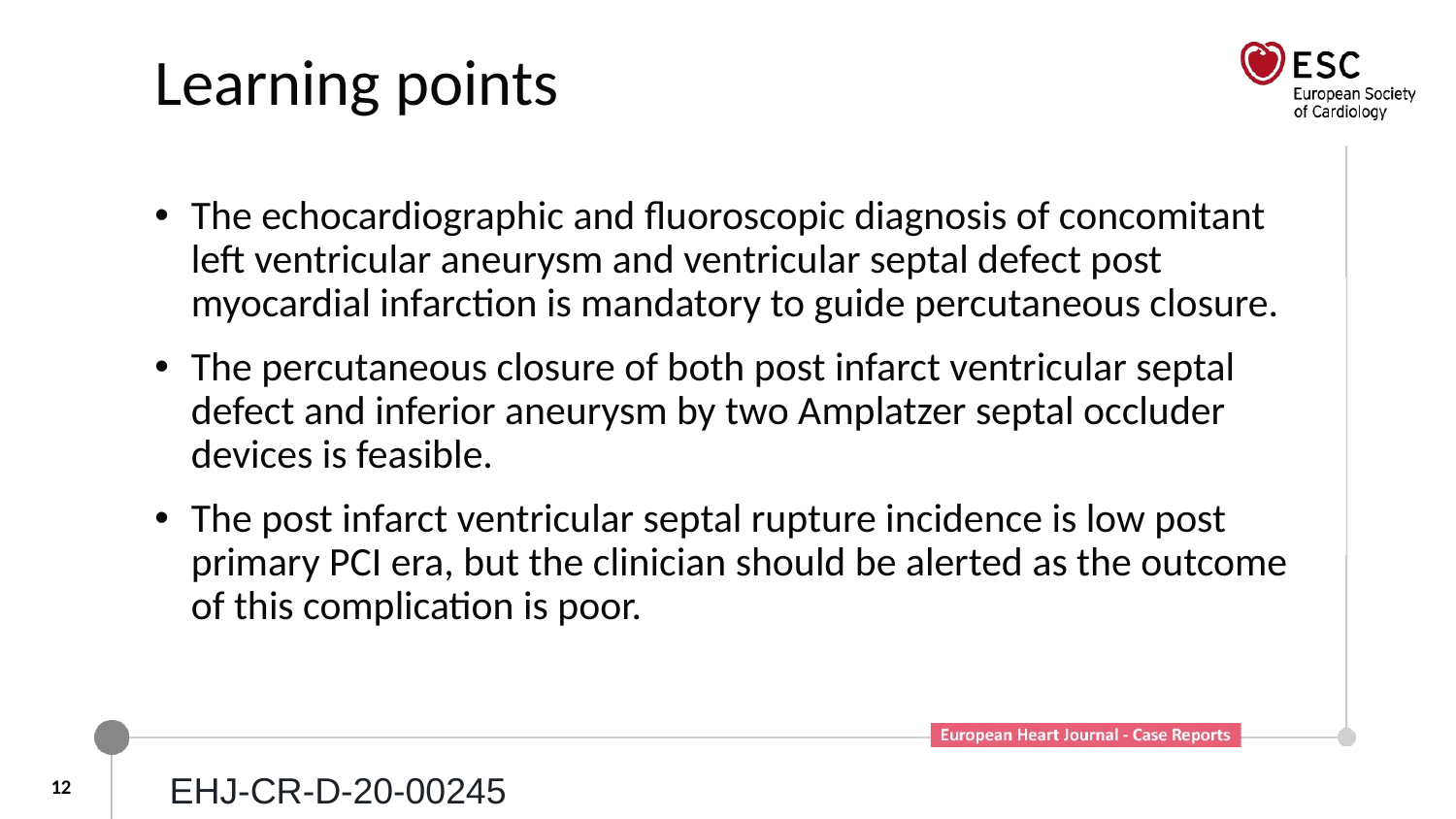

# Learning points
The echocardiographic and fluoroscopic diagnosis of concomitant left ventricular aneurysm and ventricular septal defect post myocardial infarction is mandatory to guide percutaneous closure.
The percutaneous closure of both post infarct ventricular septal defect and inferior aneurysm by two Amplatzer septal occluder devices is feasible.
The post infarct ventricular septal rupture incidence is low post primary PCI era, but the clinician should be alerted as the outcome of this complication is poor.
12
EHJ-CR-D-20-00245

## Slide 13
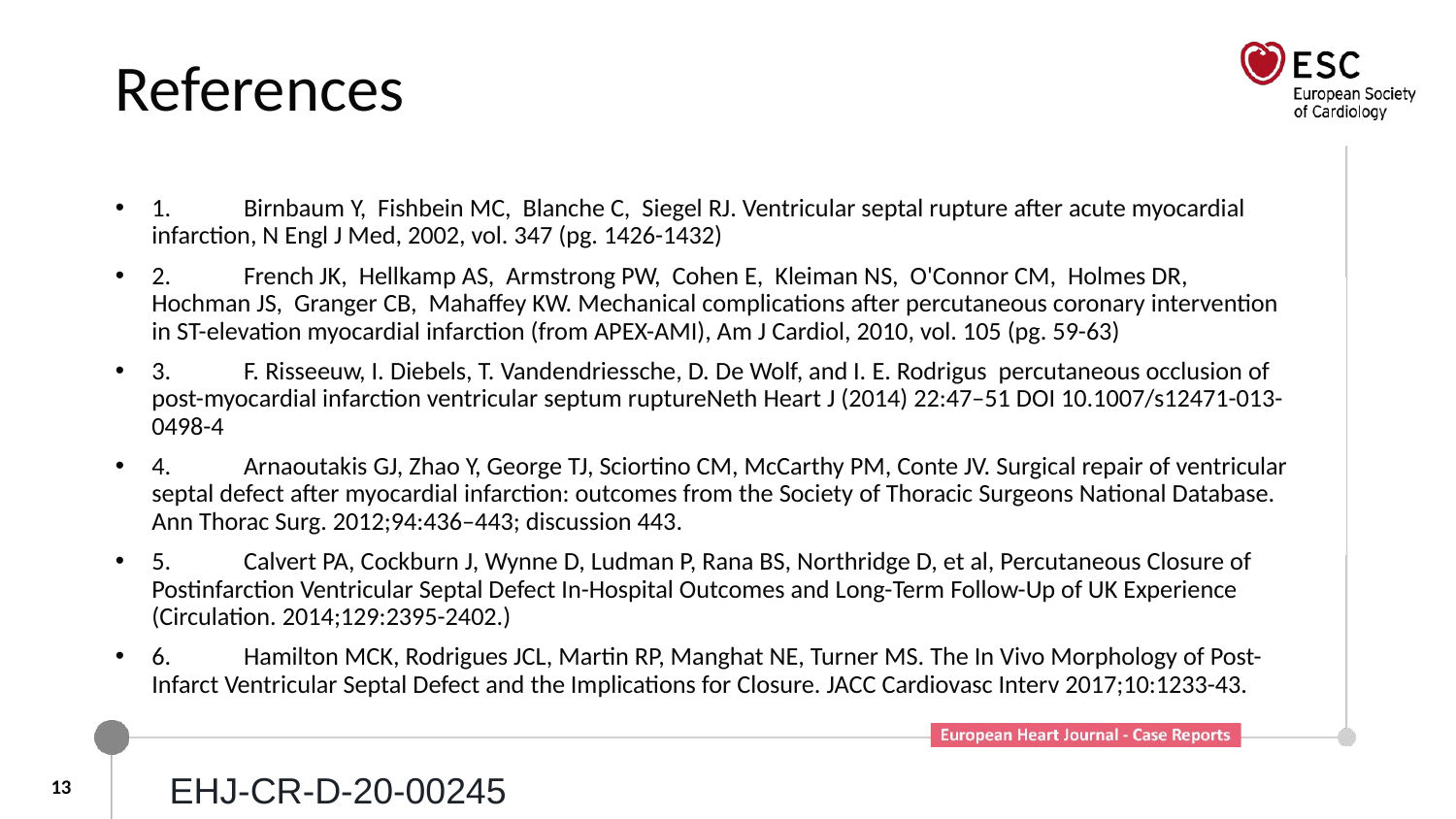

# References
1.	Birnbaum Y, Fishbein MC, Blanche C, Siegel RJ. Ventricular septal rupture after acute myocardial infarction, N Engl J Med, 2002, vol. 347 (pg. 1426-1432)
2.	French JK, Hellkamp AS, Armstrong PW, Cohen E, Kleiman NS, O'Connor CM, Holmes DR, Hochman JS, Granger CB, Mahaffey KW. Mechanical complications after percutaneous coronary intervention in ST-elevation myocardial infarction (from APEX-AMI), Am J Cardiol, 2010, vol. 105 (pg. 59-63)
3.	F. Risseeuw, I. Diebels, T. Vandendriessche, D. De Wolf, and I. E. Rodrigus percutaneous occlusion of post-myocardial infarction ventricular septum ruptureNeth Heart J (2014) 22:47–51 DOI 10.1007/s12471-013-0498-4
4.	Arnaoutakis GJ, Zhao Y, George TJ, Sciortino CM, McCarthy PM, Conte JV. Surgical repair of ventricular septal defect after myocardial infarction: outcomes from the Society of Thoracic Surgeons National Database. Ann Thorac Surg. 2012;94:436–443; discussion 443.
5.	Calvert PA, Cockburn J, Wynne D, Ludman P, Rana BS, Northridge D, et al, Percutaneous Closure of Postinfarction Ventricular Septal Defect In-Hospital Outcomes and Long-Term Follow-Up of UK Experience (Circulation. 2014;129:2395-2402.)
6.	Hamilton MCK, Rodrigues JCL, Martin RP, Manghat NE, Turner MS. The In Vivo Morphology of Post-Infarct Ventricular Septal Defect and the Implications for Closure. JACC Cardiovasc Interv 2017;10:1233-43.
13
EHJ-CR-D-20-00245
